# Associations of Serum Thiamine Levels with Blood Pressure Among Middle-Aged and Elderly Women in Eastern China

**DOI:** 10.3390/nu17132210

**Published:** 2025-07-03

**Authors:** Lijin Chen, Jingjing Lin, Xiangyu Chen, Zhimin Ma, Xiaofu Du, Meng Wang, Rong Chen, Jieming Zhong

**Affiliations:** 1 Department of Non-Communicable Disease Control and Prevention, Zhejiang Provincial Center for Disease Control and Prevention, Hangzhou 310051, China; ljch@cdc.zj.cn (L.C.); jjlin@cdc.zj.cn (J.L.); xychen@cdc.zj.cn (X.C.); mzhma@cdc.zj.cn (Z.M.); xfdu@cdc.zj.cn (X.D.); mwang@cdc.zj.cn (M.W.); 2 Department of Non-Communicable and Endemic Disease Control and Prevention, Changxing Center for Disease Control and Prevention, Changxing, Huzhou 313199, China; cxjkmbk@126.com

**Keywords:** hypertension, blood pressure, serum thiamine, vitamin B1

## Abstract

**Background**: Although B vitamins are implicated in cardiovascular regulation, the associations between serum thiamine (vitamin B1) and blood pressure (BP) remain unclear, particularly among women who are at high risk for hypertension-related complications. This study aimed to investigate relationships between serum thiamine levels and BP outcomes among middle-aged and elderly women in eastern China. **Methods**: A community-based cross-sectional study was conducted among 2015 women aged 45–69 years in Zhejiang Province, China. Serum thiamine levels were quantified using liquid chromatography tandem mass spectrometry (LC-MS/MS). Hypertension was defined as measured BP ≥ 140/90 mmHg, or current use of antihypertensive medications. Multivariate logistic and linear regression models were used to assess associations of thiamine with hypertension prevalence and BP levels, respectively. Dose–response relationships were evaluated using restricted cubic splines (RCSs). **Results**: Higher thiamine levels were significantly associated with reduced hypertension prevalence (adjusted OR per SD increase: 0.87; 95%CI: 0.77, 0.97), with RCSs confirming linear dose–response (*p*-overall < 0.05, *p*-nonlinearity > 0.05). Compared with the lowest tertile, participants in the highest thiamine tertile had a 25% lower hypertension risk. Thiamine levels also showed negative associations with systolic BP (adjusted coef: −1.51 mmHg per SD; 95% CI: −2.33, −0.68), with the participants in the highest tertile showing a 3.94 mmHg reduction (95%CI: −5.97, −1.92). No significant relationship was found for diastolic BP. **Conclusions**: Serum thiamine is inversely associated with both hypertension prevalence and systolic BP in middle-aged and elderly women. This study supports the potential of serum thiamine as a modifiable biomarker in hypertension prevention strategies, particularly among aging women.

## 1. Introduction

Hypertension is a leading modifiable cause of premature death worldwide. Hypertension affected 1.39 billion people globally in 2010, and this number could grow to 1.56 billion by 2025 [1]. The age-standardized rate of hypertension in China attained 24.7% in 2018 [2] and elevated systolic blood pressure (BP) is emerging as one of the principal contributors to death in mainland China [3]. Hypertension brings a greater burden for women than men [4], as they experience a steeper, more permanent rise in BP than men, starting as early as their thirties [5]. Female hypertensives face elevated risks of cardiac (ventricular hypertrophy, diastolic dysfunction, heart failure), vascular (arterial stiffening), and metabolic (diabetes, renal impairment) complications when compared with male counterparts [4,6,7,8]. Therefore, the need for sex-specific research is highlighted. Identifying more modifiable risk factors of hypertension in women is of great importance for public health.

Recent scientific investigations have progressively focused on the significance of B vitamins in maintaining human health [9,10,11]. Thiamine (vitamin B1), an indispensable water-soluble micronutrient, serves critical metabolic functions as (1) a carbohydrate metabolism cofactor, (2) a lipid oxidation mediator, and (3) a protein catabolism facilitator [12,13,14]. Endogenous production being absent, this nutrient must be derived from the diet, with rich sources encompassing animal proteins (pork, fish), plant-based foods (nuts, whole grains), and certain vegetables like asparagus [15,16]. Thiamine deficiency often results from a series of predisposing conditions, including insufficient dietary intake, impaired intestinal absorption, elevated metabolic needs, or enhanced renal excretion. Historically, individuals with chronic alcoholism, acquired immune deficiency syndrome, and cancer have constituted the primary high-risk people for this nutritional deficit [17]. Physiological states characterized by heightened metabolic requirements—including life stages (lactation, pregnancy), endocrine disorders (hyperthyroidism, diabetes), and pathological conditions (critical illness, infection, end-stage renal disease)—demonstrate significantly increased susceptibility to thiamine deficiency [18]. Additionally, post-bariatric surgery patients are more likely to suffer from thiamine deficiency due to the vitamin’s low bioavailability [19]. Accumulating research demonstrates associations between thiamine insufficiency and diverse negative health consequences, encompassing mortality risk, cardiometabolic disorders, glucose metabolism dysregulation, heart failure, obesity, dyslipidemia, angina, myocardial infarction, anemia, and depression [20,21,22,23,24,25,26,27,28,29,30]. Current evidence regarding thiamine’s potential role in hypertension pathogenesis remains scarce and inconsistent. For instance, research employing spontaneously hypertensive rats has revealed that thiamine repletion induced significant blood pressure reduction [31], whereas an epidemiological study reported a U-shaped association of thiamine consumption (measured by interview) with hypertension risk [32]. Notably, the potential connection between serum thiamine and BP in women has yet to be revealed.

To bridge the above knowledge gaps, this study aims to evaluate the associations of serum thiamine with BP outcomes among middle-aged and elderly women in a community-based population.

## 2. Materials and Methods

### 2.1. Study Design and Population

The present cross-sectional study was conducted between March and May 2019. A total of 2015 female participants in Heping, Lijiaxiang and Lincheng community in Changxing, Zhejiang Province, China, were recruited through convenience sampling. Eligible participants met the following criteria: (1) were community-dwelling adults aged 45–69 years; (2) had the physical and cognitive capacity to undergo comprehensive assessments; (3) and were non-pregnant and of non-lactating status.

All the participants were asked to complete standardized health assessments including anthropometric measurements, blood sampling and BP measurements. Participants also completed face-to-face questionnaire surveys with questions regarding demographic characteristics, lifestyle factors, and medical history information.

**Figure 1:**
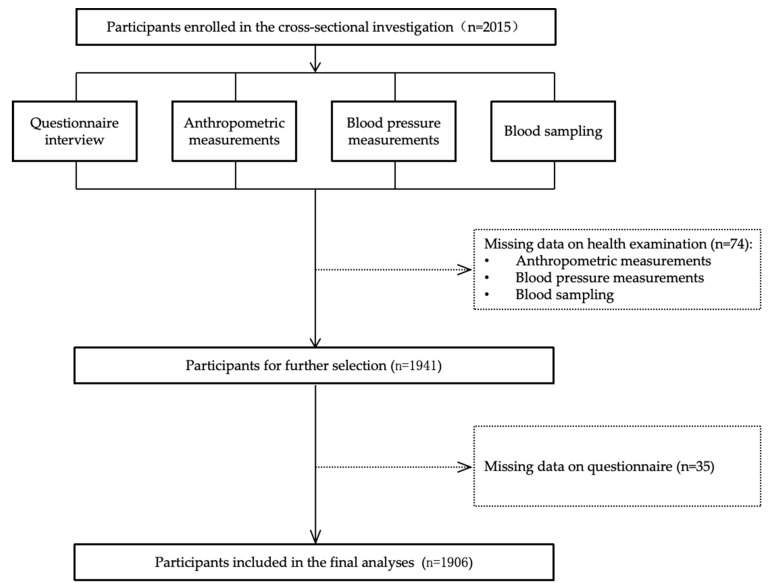
Flowchart of data collection and participant enrollment through final analysis.

The analysis excluded individuals lacking information on health examination (age, weight, height, BP, blood biomarkers, n = 74) or questionnaire (educational level, menopausal status, drinking frequency, current medications, n = 35). After the exclusion, 1906 female subjects remained eligible for the final analyses. The flowchart of data collection and participant enrollment through to final analysis is presented in Figure 1.

### 2.2. Anthropometric Measurements and Questionnaire Survey

Trained personnel from CDC-affiliated facilities and community health centers implemented standardized data collection protocols. Anthropometric measurements were obtained following standardized protocols, with height (nearest 0.1 cm) and weight (nearest 0.01 kg) being recorded with participants wearing lightweight attire without footwear. BMI was derived using the standard formula (weight [kg]/height^2^ [m]). Triplicate measurements were averaged for final values.

All participants completed structured face-to-face questionnaire interviews conducted by trained personnel. The questionnaire collected detailed information on demographic characteristics, menopausal status, drinking frequency, self-reported disease history, and current medication use. Drinking frequency was classified as never, occasionally, and frequently. Educational level was categorized as without any formal schooling, primary school, junior high school, senior high school, college and above. It was included as a validated proxy for socioeconomic status (SES), a potential confounder in the relationship between nutritional biomarkers and health outcomes. SES may influence serum thiamine levels through dietary quality, and hypertension risk via healthcare access or protective environmental factors. Chronic kidney disease (CKD) status (yes/no) was based on self-reported physician diagnosis. Use of thiamine-specific or B-complex supplements within the past 2 weeks (yes/no) was recorded.

### 2.3. Blood Pressure Measurement and Definition of Hypertension

BP was measured at least twice following a 5 min seated rest period, using an electronic BP monitor (OMRON Corporation J7136, Kyoto, Japan). The mean value of the two recorded measurements was utilized. Hypertensive status was determined by meeting threshold BP values (≥140/90 mmHg) or current antihypertensive treatment.

### 2.4. Laboratory Measurements and Definition of Disease

After an overnight fast of at least 12 h, venous blood specimens were collected from all participants. Laboratory assessments included thiamine, total cholesterol (TC), triglyceride (TG), low density lipoprotein (LDL-c), high-density lipoprotein cholesterol (HDL-c), and fasting plasma glucose (FPG). All laboratory examinations were performed following standardized laboratory protocols. Thiamine levels in serum were determined by high-performance liquid chromatography coupled with tandem mass spectrometry (HPLC-MS/MS) [33]. Participants were classified into 3 groups based on thiamine tertiles.

The operational definition of dyslipidemia encompassed any of the following criteria: TC levels ≥ 6.22 mmol/L, TG levels ≥ 2.26 mmol/L, LDL-c levels ≥ 4.14 mmol/L, HDL-c levels < 1.04 mmol/L, or current lipid-lowering medications [34]. Abnormal blood glucose was defined as FPG ≥ 7.0 mmol/or the use of antidiabetic medications [35].

### 2.5. Statistical Analysis

Continuous variables were expressed as mean ± standard deviation (SD) for normally distributed data or median (interquartile range, IQR) for non-normally distributed parameters. Categorical variables were displayed using frequency (percentage). Group comparisons between hypertensive and normotensive participants were performed using appropriate statistical tests: student’s *t* test for normally distributed data, Wilcoxon rank-sum test for non-normally distributed data, and the Chi-square (χ^2^) test for categorical data. *p* for trend was calculated across thiamine tertiles. Blood pressure was clinically recorded as integers. During statistical analysis, computational procedures inherently generate decimal values. To preserve analytical precision and ensure consistency with other continuous variables (e.g., age, BMI), all statistical outputs retain necessary decimals.

Both crude and adjusted logistic regression analyses were performed to examine the relationship between hypertension prevalence and serum thiamine levels, analyzed as both continuous and categorical (tertile) variables. In addition, the dose–response association of hypertension prevalence with serum thiamine concentration was visualized by plotting restricted cubic splines (RCSs) [36], with 3 knots (10th, 30th, 90th percentiles of serum thiamine concentration). *p*-overall and *p*-nonlinearity were reported. Both unadjusted and adjusted linear regression analyses were used to explore the relationships of systolic and diastolic BP with serum thiamine concentration and tertiles.

For all previously described regression approaches, progressive adjustment strategies were employed. Model 1 (crude) examined the unadjusted association; Model 2 added adjustments for demographic and lifestyle factors including age, menopausal status, BMI, educational level and drinking status; finally, Model 3 further incorporated dyslipidemia and abnormal blood glucose to evaluate potential confounding from cardiometabolic comorbidities on the serum thiamine–hypertension relationship. To determine whether the associations between thiamine and BP were influenced by the inclusion of participants taking antihypertensive medications, a sensitivity analysis was performed that excluded participants taking antihypertensive medications (n = 701). The univariate and multivariate models were re-run in this medication-free subgroup. Meanwhile, to evaluate potential confounding from CKD, a sensitivity analysis excluding participants with CKD (n = 7) was conducted. Similarly, a separate sensitivity analysis was undertaken that excluded recent users of thiamine or B-complex supplements (n = 5) in order to address potential confounding. Statistical significance was defined as a two-tailed *p*-value below 0.05. All data analyses were performed using STATA 14 for Windows (StataCorp LP, College Station, TX, USA).

## 3. Results

### 3.1. General Characteristics of Participants

A total of 1906 female participants were included in the analysis, among whom 1166 had hypertension. The study population had an average age of 59.93 ± 6.64 years, and 90.56% were postmenopausal women. The median serum thiamine was 1.75 (IQR: 1.31–2.32) ng/mL. Comparative characteristics between hypertensive and non-hypertensive groups are summarized in Table 1. Hypertensive participants were significantly older, more likely to be postmenopausal, and exhibited higher BMI values and lower educational attainment than normotensive participants. Moreover, participants with hypertension demonstrated significantly poorer lipid metabolism parameters, including elevated triglyceride and LDL cholesterol levels coupled with reduced HDL cholesterol, along with higher fasting plasma glucose compared with normotensive individuals. The serum thiamine concentration was lower in participants with hypertension than without (all *p* < 0.05).

To further examine the relationship between thiamine and hypertension, Table 2 presents BP levels and hypertension prevalence across thiamine tertiles. Elevated thiamine tertiles showed graded associations with decreased hypertension prevalence and systolic BP (both *p* value for trend < 0.05). No differences in diastolic BP were observed among thiamine tertiles (*p* value for trend > 0.05).

### 3.2. Associations Between Serum Thiamine and Hypertension Prevalence in Women

The unadjusted and adjusted associations between serum thiamine and hypertension prevalence are presented in Table 3. Every SD increase in thiamine concentration was negatively associated with hypertension (adjusted OR (95%CI): 0.87 (0.77,0.97), *p* = 0.012). Compared with the lowest tertile of thiamine, the highest tertile was negatively associated with hypertension (adjusted OR (95%CI): 0.75 (0.59,0.95), *p* = 0.016). The observed associations remained significant after sequential adjustments for covariates (all *p* < 0.05).

### 3.3. Dose–Response Relationship Between Serum Thiamine and Hypertension Prevalence in Women

**Figure 2:**
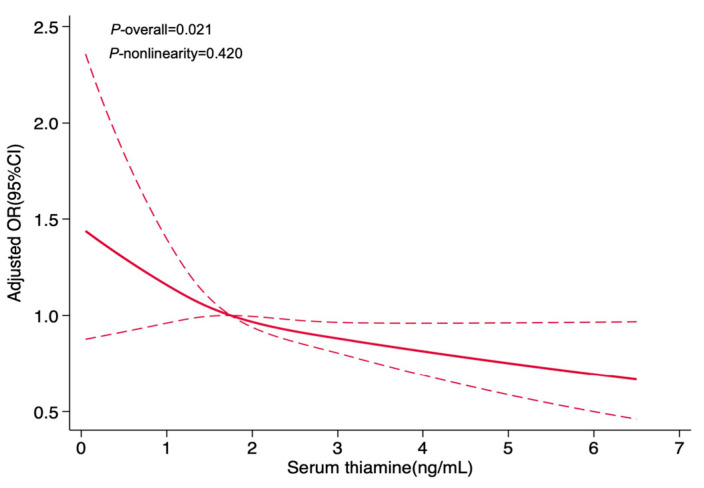
Association between serum thiamine and hypertension in women, allowing for nonlinear effects, with 95%CIs. Adjusted for age, menopausal status, BMI, educational level, drinking status, dyslipidemia and abnormal blood glucose. The dashed line represents the upper and lower bounds of 95% CI around the predicted odds ratios. Abbreviations: OR, odds ratio; CI, confidence interval.

To further investigate the dose–response relationship between serum thiamine and hypertension risk, RCS was performed (Figure 2). Figure 2 revealed a linear relationship between thiamine and hypertension (*p*-overall < 0.05, *p*-nonlinearity > 0.05), with hypertension risk decrease observed alongside rising thiamine levels.

### 3.4. Associations Between Serum Thiamine and BP in Women

The unadjusted and adjusted coefficients and 95% CIs for the associations between thiamine and systolic BP are presented in Table 4. Every SD increase in thiamine concentration was negatively associated with systolic BP (adjusted coef (95%CI): −1.51 (−2.33, −0.68), *p* < 0.001). Compared with the lowest tertile of thiamine, the highest tertile was negatively associated with systolic BP (adjusted coef (95%CI): −3.94 (−5.97, −1.92), *p* < 0.001). The observed associations remained significant after sequential adjustments for covariates (all *p* < 0.05).

Table 5 presents the unadjusted and adjusted coefficients and 95% CIs for the association between serum thiamine and diastolic BP. Every SD increase in thiamine concentration was negatively associated with diastolic BP in Model 2 and Model 3 (adjusted coef (95%CI): −0.53 (−0.95, −0.10), *p* =0.015). No significant association between thiamine tertiles and diastolic BP was observed (all coef < 0, all *p* > 0.05).

### 3.5. Sensitivity Analysis

**Figure 3:**
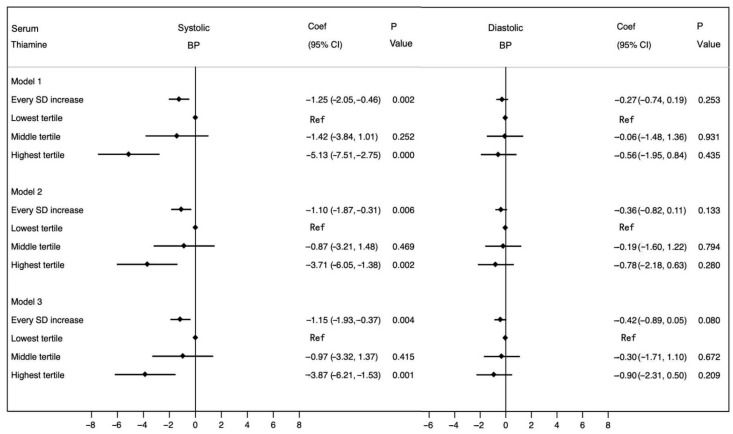
Unadjusted and multivariable-adjusted coefficients and 95%CIs for BP after excluding participants taking antihypertensive medications (n = 1205). Model 1: crude model. Model 2: adjusted for age, menopausal status, BMI, educational level and drinking status. Model 3: adjusted for age, menopausal status, BMI, educational level, drinking status, dyslipidemia and abnormal blood glucose. Abbreviations: coef, coefficient; CI, confidence interval; SD, standard deviation; BP, blood pressure; Ref, reference.

To assess whether the negative associations between thiamine and BP were affected by current use of antihypertensive medications, we conducted a sensitivity analysis by excluding participants taking antihypertensive medications. Final analyses included a total of 1205 female subjects. Both univariate and multivariate linear regression models were performed (Figure 3), and the results revealed that the associations between serum thiamine and systolic BP remained statistically significant and directionally consistent with the primary findings (all coef < 0, all *p* < 0.05). No significant association was observed for diastolic BP.

**Figure A1:**
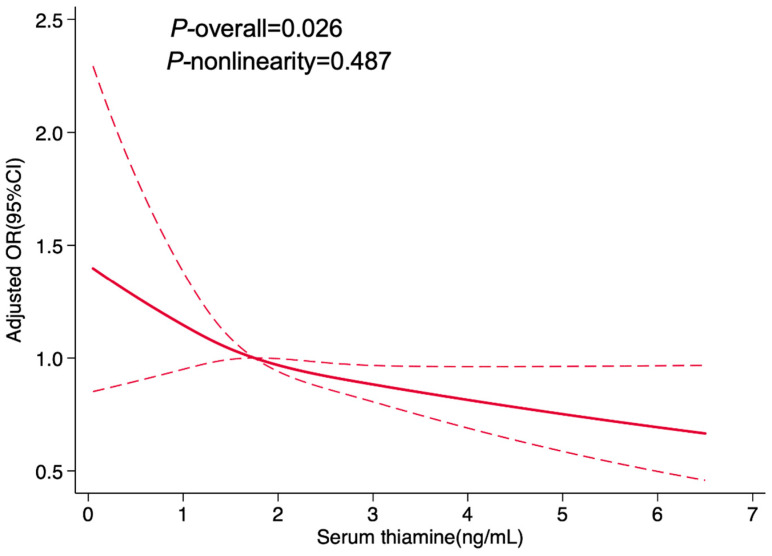
Association between serum thiamine and hypertension in women after excluding participants with CKD, allowing for nonlinear effects, with 95%CIs. Adjusted for age, menopausal status, BMI, educational level, drinking status, dyslipidemia and abnormal blood glucose. The dashed line represents the upper and lower bounds of 95% CI around the predicted odds ratios. Abbreviations: OR, odds ratio; CI, confidence interval.

Meanwhile, to evaluate potential confounding from CKD, a sensitivity analysis excluding participants with CKD (n = 7) was conducted. As shown in Table A1 and Table A2, serum thiamine maintained significant negative associations with hypertension prevalence and systolic BP, respectively. Figure A1 demonstrated a linear relationship between serum thiamine and hypertension prevalence. These findings remain consistent with the primary analysis. Similarly, a separate sensitivity analysis excluding recent thiamine/B-complex supplement users (n = 5) yielded virtually identical results.

## 4. Discussion

In this community-based cross-sectional study, we found that higher serum thiamine levels are significantly and linearly associated with reduced hypertension prevalence. Inverse associations between serum thiamine levels and blood pressure are more pronounced for systolic BP than for diastolic BP and are not affected by antihypertensive medication use.

Previous studies examining the thiamine–hypertension association have primarily relied on 24 h dietary recall to estimate thiamine intake [21,32,37,38,39], a method potentially limited by recall bias and measurement errors. Furthermore, the observed associations might be confounded by thiamine’s heat susceptibility during cooking and interindividual variations in bioavailability [40,41]. In contrast, serum thiamine levels act as a more objective biomarker of thiamine status, integrating both dietary exposure and physiological utilization. However, studies adopting serum thiamine to examine its associations with hypertension were not found. To address the gaps in knowledge, this study was the first to investigate the associations between serum thiamine and BP in a Chinese female population.

Overall, we found negative associations between serum thiamine with systolic BP and hypertension prevalence. The findings align with previous studies. Using dietary data from the US National Health and Nutrition Examination Survey (NHANES), Wen and colleagues identified a significant inverse relationship between thiamine consumption and the prevalence of hypertension. Thiamine intake was estimated by 24 h recall. A gradual trend toward decreasing hypertension prevalence with increasing dietary vitamin B1 intake was also found in this study [21]. An observational study based on the Korean National Health and Nutrition Examination Survey (KNHANES) supported similar findings [22]. In an animal experiment conducted by Tanaka et al., After 10 weeks of 0.2% thiamine supplementation via drinking water, four-week-old SHR demonstrated significant systolic blood pressure lowering when compared with untreated controls [31]. In our study, RCS analysis showed a linear association between serum thiamine with hypertension. However, nonlinear associations are reported in an existing study [32]. This inconsistency might be explained by the differing estimates of thiamine used [42]. We used serum thiamine data, while previous studies adopted dietary data.

The observed association between thiamine and hypertension was supported by the established pathophysiological mechanisms. In a genetic investigation combining extreme phenotype analysis with genome-wide pooled data, Zhang and colleagues identified a connection between a thiamine transporter (encoded by the SLC35F3 gene variant) and abnormalities in heart function and autonomic regulation. Individuals carrying two copies of the risk-linked allele showed reduced thiamine levels in red blood cells and exhibited cardiovascular patterns linked to thiamine insufficiency—such as increased heart stroke volume, lower vascular resistance, and heightened blood pressure responses—indicating a potential role of this transporter in the pathogenesis of hypertension [43]. Furthermore, the SHR study demonstrated thiamine’s antihypertensive effects through transcriptional regulation of renin–angiotensin system components, as evidenced by altered mRNA expression profiles [31]. Furthermore, supplemental thiamine has also been shown to boost endothelial-dependent vascular relaxation and inhibit the advancement of atherosclerosis [40]. These studies have suggested that thiamine is associated with the mechanism of BP control. As a cofactor of transketolase, thiamine deficiency reduces the clearance of glycolytic metabolites (e.g., glyceraldehyde-3-phosphate), leading to the accumulation of advanced glycation end-products (AGEs) that promote collagen cross-linking in arterial walls [14]. Given that arterial stiffness predominantly increases systolic BP with minimal diastolic effect [44], this mechanism may explain the stronger association with SBP than DBP observed in our study. This mechanism also explains sex-specific BP trajectories—pulse wave analysis revealed that middle-aged and elderly women exhibited significantly higher large artery stiffness than men, leading to elevated systolic BP but reduced diastolic BP [45,46]. Consequently, the prevalence of isolated diastolic hypertension in women aged ≥40 years was found to be only 3.1% [47], with isolated systolic hypertension overwhelmingly predominating. These findings emphasize the importance of sufficient thiamine for the prevention of systolic hypertension in this high-risk population. Given the persistent inverse association between thiamine levels and hypertension prevalence, we propose that nutritional interventions be prioritized for middle-aged and older women with low thiamine levels. Increasing the intake of natural dietary sources rich in thiamine—such as whole grains, legumes, and lean meats—can progressively improve thiamine nutritional status. For high-risk individuals who struggle to meet requirements through diet alone, thiamine supplementation under medical guidance may provide additional benefits. Future randomized controlled trials are needed to systematically evaluate the dose–response relationship and long-term efficacy of thiamine supplementation for BP control. Such studies will provide crucial evidence for developing precise nutritional strategies.

While the wide age range (45–69 years) encompasses heterogeneous physiological stages, we mitigated this by adjusting for age as a continuous covariate and menopausal status, which retains full informational value and enhances statistical efficiency in modeling age-related variations. To further assess the robustness of our findings, we performed three sensitivity analyses. First, we excluded participants taking antihypertensive medications and confirmed that the relationship between thiamine and BP was independent of pharmacologic treatment. Second, given that thiamine elimination occurs primarily via renal excretion [48], we excluded participants with CKD (n = 7) and found that the inverse associations persisted, indicating that altered renal excretion did not confound our findings. Finally, although serum thiamine integrates dietary intake, supplementation, and metabolism, supplement users may exhibit distinct health behaviors (e.g., better health consciousness) that influence hypertension risk. To address potential confounding, we excluded recent users of thiamine/B-complex supplements (n = 5) and observed that the negative associations were maintained; this suggests that our results reflect biological relationships independent of such supplement-related behaviors. Collectively, these analyses support the robustness of the observed inverse link.

This study has several strengths. First, sex-specific associations between thiamine and hypertension were explored. Second, serum thiamine was used to measure thiamine levels in the body, which is more objective than the self-reporting of thiamine intake. Third, our analysis adjusted for a series of confounders including age, menopausal status, BMI, educational level, drinking status, dyslipidemia and abnormal blood glucose, which may impact the associations between serum thiamine and hypertension. Additionally, the effect of antihypertensive medications was accounted for in the analysis.

Several study limitations merit acknowledgment. First, its cross-sectional design prevents the establishment of causal relationships. Second, serum thiamine levels represent short-term thiamine intake. Further studies using the erythrocyte transketolase activity assay (ETKA) are needed to evaluate long-term thiamine status. ETKA assesses transketolase activity as an indicator of whole-body thiamine levels [42]. Third, data on hormone replacement therapy (HRT) use were not collected. Although we adjusted for menopausal status to account for related biological changes, residual confounding by unmeasured HRT use cannot be entirely excluded. Fourth, this study did not collect data on diuretic use. Although sensitivity analyses that excluded hypertensive medication users or chronic kidney disease patients (covering most diuretic exposure) showed unchanged results, residual confounding from unmeasured diuretic use cannot be entirely ruled out. Finally, information on antihypertensive medication use was limited to current treatment status (yes/no). Specific drug classes (e.g., ACE inhibitors, calcium channel blockers, diuretics) were not recorded. Further studies are needed to provide deeper and more detailed findings.

## 5. Conclusions

In conclusion, this community-based cross-sectional study demonstrates that higher serum thiamine levels are significantly and linearly associated with reduced hypertension prevalence. Inverse associations between serum thiamine levels and blood pressure are more pronounced for systolic BP than for diastolic BP and are not affected by antihypertensive medication use. These findings highlight the potential role of thiamine in blood pressure regulation and suggest that serum thiamine could be a modifiable biomarker and intervention target for hypertension prevention in middle-aged and elderly women. Future longitudinal and interventional studies are warranted to explore the causal relationships.

## Figures and Tables

**Table 1:** Characteristics of participants with and without hypertension (n = 1906).

Variables	Overall (n = 1906)	Participants Without Hypertension (n = 740)	Participants with Hypertension (n = 1166)	t/χ^2^/z	*p*
Age, mean ± SD, years	59.93 ± 6.64	58.47 ± 6.94	60.86 ± 6.28	60.34 ^a^	<0.001
Educational level, n (%)				24.17 ^b^	<0.001
Without any formal schooling	858 (45.02)	293 (39.59)	565 (48.46)		
Primary school	634 (33.26)	251 (33.92)	383 (32.85)		
Junior high school	351 (18.42)	163 (22.03)	188 (16.12)		
Senior high school	57 (2.99)	28 (3.78)	29 (2.49)		
College and above	6 (0.31)	5 (0.68)	1 (0.09)		
BMI, mean ± SD, kg/m^2^	24.45 ± 3.59	23.66 ± 3.07	24.95 ± 3.79	61.01 ^a^	<0.001
Menopausal status, n (%)				11.52 ^b^	0.001
Pre-menopausal	180 (9.44)	91 (12.30)	89 (7.63)		
Post-menopausal	1726 (90.56)	649 (87.70)	1077 (92.37)		
Drinking status, n (%)				0.12 ^b^	0.941
Never	1642 (86.15)	635 (85.81)	1007 (86.36)		
Occasionally	125 (6.56)	50 (6.76)	75 (6.43)		
Frequently	139 (7.29)	55 (7.43)	84 (7.20)		
TC, mean ± SD, mmol/L	5.04 ± 0.95	5.03 ± 1.00	5.04 ± 0.93	0.12 ^a^	0.728
TG, median (IQR), mmol/L	1.38 (1.01–1.94)	1.28 (0.91–1.76)	1.46 (1.08–2.06)	−6.97 ^c^	<0.001
LDL-c, mean ± SD, mmol/L	2.89 ± 0.72	2.84 ± 0.68	2.92 ± 0.74	6.31 ^a^	0.012
HDL-c, median (IQR), mmol/L	1.60 (1.36–1.84)	1.67 (1.43–1.90)	1.55 (1.33–1.8)	5.91 ^c^	<0.001
Lipid-lowering medications use, n (%)	44 (18.49)	8 (13.79)	36 (20.00)	1.22 ^b^	0.749
Dyslipidemia, n (%)	525 (27.54)	159 (21.49)	366 (31.39)	22.24 ^b^	<0.001
FPG, median (IQR), mmol/L	5.48 (5.07–6.10)	5.37 (4.98–5.87)	5.56 (5.13–6.23)	−5.93 ^c^	<0.001
Antidiabetic medication use, n (%)	186 (83.41)	60 (83.33)	126 (83.44)	0.00 ^b^	0.983
Abnormal blood glucose, n (%)	296 (15.53)	88 (11.89)	208 (17.84)	12.20 ^b^	<0.001
Thiamine, median (IQR), ng/mL	1.75 (1.31–2.32)	1.81 (1.38–2.36)	1.73 (1.27–2.28)	3.04 ^c^	0.002
Thiamine tertiles, n (%)				10.23 ^b^	0.006
Lowest	625 (32.79)	216 (29.19)	409 (35.08)		
Middle	639 (33.53)	246 (33.24)	393 (33.70)		
Highest	642 (33.68)	278 (37.57)	364 (31.22)		
Systolic BP, mean ± SD, mmHg	139.87 ± 19.08	124.20 ± 10.68	149.81 ± 16.38	1424.56 ^a^	<0.001
Diastolic BP, mean ± SD, mmHg	81.24 ± 10.60	75.50 ± 8.04	84.89 ± 10.41	435.89 ^a^	<0.001

^a^ Student’s *t*-test; ^b^ Chi-square test; ^c^ Wilcoxon rank-sum test. Abbreviations: BMI, body mass index; TC, total cholesterol; TG, triglyceride; LDL-C, low-density lipoprotein cholesterol; HDL-C, high-density lipoprotein cholesterol; BP, blood pressure; SD, standard deviation; IQR, interquartile range.

**Table 2:** Hypertension prevalence and BP by thiamine tertiles (n = 1906).

Hypertension Prevalence or BP	Thiamine Tertiles			*p* For Trend
	Lowest (n = 625)	Middle (n = 639)	Highest (n = 642)	
Hypertension, n (%)	409 (65.44)	393 (61.50)	364 (56.70)	0.001
Systolic BP, mean ± SD, mmHg	141.97 ± 18.57	140.80 ± 19.05	136.89 ± 19.27	<0.001
Diastolic BP, mean ± SD, mmHg	81.45 ± 10.88	81.55 ± 10.67	80.73 ± 10.24	0.314

Abbreviations: BP, blood pressure; SD, standard deviation.

**Table 3:** Unadjusted and multivariable-adjusted ORs and 95% CIs for the association between serum thiamine and hypertension prevalence (n = 1906).

Serum Levels of Thiamine	n	Model 1			Model 2			Model 3		
		OR	95% CI	*p*	OR	95% CI	*p*	OR	95% CI	*p*
Continuous variable										
Every SD increase	1906	0.86	0.77, 0.97	0.011	0.88	0.78, 0.98	0.019	0.87	0.77, 0.97	0.012
Categorical variable										
Lowest tertile	625	1.00	Ref	-	1.00	Ref	-	1.00	Ref	-
Middle tertile	639	0.84	0.67, 1.06	0.146	0.87	0.69, 1.11	0.266	0.86	0.68, 1.09	0.219
Highest tertile	642	0.68	0.54, 0.85	0.001	0.77	0.60, 0.97	0.028	0.75	0.59, 0.95	0.016

Model 1: crude model. Model 2: adjusted for age, menopausal status, BMI, educational level and drinking status. Model 3: adjusted for age, menopausal status, BMI, educational level, drinking status, dyslipidemia and abnormal blood glucose. Abbreviations: OR, odds ratio; CI, confidence interval; SD, standard deviation; Ref, reference.

**Table 4:** Unadjusted and multivariable-adjusted coefficients and 95% CIs for the association between serum thiamine and systolic BP (n = 1906).

Serum Levels of Thiamine	n	Model 1			Model 2			Model 3		
		Coef	95% CI	*p*	Coef	95% CI	*p*	Coef	95% CI	*p*
Continuous variable										
Every SD increase	1906	−1.61	−2.46, −0.75	<0.001	−1.46	−2.29, −0.64	0.001	−1.51	−2.33, −0.68	<0.001
Categorical variable										
Lowest tertile	625	0.00	Ref	-	0.00	Ref	-	0.00	Ref	-
Middle tertile	639	−1.17	−3.27, 0.92	0.272	−0.68	−2.70, 1.33	0.506	−0.72	−2.73, 1.29	0.485
Highest tertile	642	−5.08	−7.17, −2.99	<0.001	−3.85	−5.88, −1.82	<0.001	−3.94	−5.97, −1.92	<0.001

Model 1: crude model. Model 2: adjusted for age, menopausal status, BMI, educational level and drinking status. Model 3: adjusted for age, menopausal status, BMI, educational level, drinking status, dyslipidemia and abnormal blood glucose. Abbreviations: coef, coefficient; CI, confidence interval; SD, standard deviation; BP, blood pressure; Ref, reference.

**Table 5:** Unadjusted and multivariable-adjusted coefficients and 95% CIs for the association between serum thiamine and diastolic BP (n = 1906).

Serum Levels of Thiamine	n	Model 1			Model 2			Model 3		
		Coef	95% CI	*p*	Coef	95% CI	*p*	Coef	95% CI	*p*
Continuous variable										
Every SD increase	1906	−0.41	−0.84, 0.02	0.059	−0.47	−0.90, −0.05	0.029	−0.53	−0.95, −0.10	0.015
Categorical variable										
Lowest tertile	625	0.00	Ref	/	0.00	Ref	/	0.00	Ref	/
Middle tertile	639	0.10	−1.07, 1.27	0.866	0.01	−1.15, 1.17	0.984	−0.09	−1.25, 1.07	0.880
Highest tertile	642	−0.73	−1.89, 0.44	0.223	−0.90	−2.07, 0.27	0.132	−1.02	−2.19, 0.15	0.087

Model 1: crude model. Model 2: adjusted for age, menopausal status, BMI, educational level and drinking status. Model 3: adjusted for age, menopausal status, BMI, educational level, drinking status, dyslipidemia and abnormal blood glucose. Abbreviations: coef, coefficient; CI, confidence interval; SD, standard deviation; BP, blood pressure; Ref, reference.

## Data Availability

The data presented in this study are available on request from the corresponding author (the data are not publicly available due to privacy restrictions).

## Abbreviations

The following abbreviations are used in this manuscript:

BP	Blood pressure
LC-MS/MS	Liquid chromatography tandem mass spectrometry
RCS	Restricted cubic spline
SD	Standard deviation
IQR	Interquartile range
OR	Odds ratio
CI	Confidence interval
Coef	Coefficient
BMI	Body mass index
SES	Socioeconomic status
TC	Cholesterol
TG	Triglyceride
LDL-c	Low density lipoprotein
HDL-c	High-density lipoprotein cholesterol
FPG	Fasting plasma glucose
Ref	Reference
NHANES	National Health and Nutrition Examination Survey
KNHANES	Korean National Health and Nutrition Examination Survey
SHR	Spontaneous hypertensive rats
ETKA	Erythrocyte transketolase activity assay
HRT	Hormone replacement therapy

## Appendix A

**Table A1:** Unadjusted and multivariable-adjusted ORs and 95% CIs for the association between serum thiamine and hypertension prevalence after excluding participants with CKD (n = 1899).

Serum Levels of Thiamine	n	Model 1			Model 2			Model 3		
		OR	95% CI	*p*	OR	95% CI	*p*	OR	95% CI	*p*
Continuous variable										
Every SD increase	1899	0.88	0.79, 0.97	0.012	0.89	0.80, 0.98	0.020	0.88	0.80, 0.97	0.013
Categorical variable										
Lowest tertile	622	1.00	Ref	-	1.00	Ref	-	1.00	Ref	-
Middle tertile	637	0.86	0.68, 1.08	0.188	0.89	0.70, 1.13	0.336	0.88	0.69, 1.11	0.280
Highest tertile	640	0.70	0.56, 0.88	0.002	0.77	0.61, 0.98	0.035	0.75	0.59, 0.96	0.021

Model 1: crude model. Model 2: adjusted for age, menopausal status, BMI, educational level and drinking status. Model 3: adjusted for age, menopausal status, BMI, educational level, drinking status, dyslipidemia and abnormal blood glucose. Abbreviations: OR, odds ratio; CI, confidence interval; SD, standard deviation; Ref, reference.

**Table A2:** Unadjusted and multivariable-adjusted coefficient and 95% CIs for the association between serum thiamine and systolic BP after excluding participants with CKD (n = 1899).

Serum Levels of Thiamine	n	Model 1			Model 2			Model 3		
		Coef	95% CI	*p*	Coef	95% CI	*p*	Coef	95% CI	*p*
Continuous variable										
Every SD increase	1899	−1.44	−2.21, −0.67	<0.001	−1.31	−2.05, −0.57	0.001	−1.35	−2.09, −0.61	<0.001
Categorical variable										
Lowest tertile	622	0.00	Ref	-	0.00	Ref	-	0.00	Ref	-
Middle tertile	637	−1.12	−3.22, 0.98	0.295	−0.62	−2.64, 1.40	0.548	−0.66	−2.67, 1.36	0.524
Highest tertile	640	−5.06	−7.15, −2.96	<0.001	−3.81	−5.84, −1.77	<0.001	−3.90	−5.93, −1.86	<0.001

Model 1: crude model. Model 2: adjusted for age, menopausal status, BMI, educational level and drinking status. Model 3: adjusted for age, menopausal status, BMI, educational level, drinking status, dyslipidemia and abnormal blood glucose. Abbreviations: coef, coefficient; CI, confidence interval; SD, standard deviation; BP, blood pressure; Ref, reference.
